# Epigenetic Inactivation of Heparan Sulfate (Glucosamine) 3-*O*-Sulfotransferase 2 in Lung Cancer and Its Role in Tumorigenesis

**DOI:** 10.1371/journal.pone.0079634

**Published:** 2013-11-12

**Authors:** Jung-Ah Hwang, Yujin Kim, Seung-Hyun Hong, Jieun Lee, Yong Gu Cho, Ji-Youn Han, Young-Ho Kim, Joungho Han, Young Mog Shim, Yeon-Su Lee, Duk-Hwan Kim

**Affiliations:** 1 Cancer Genomics Branch, Research Institute, National Cancer Center, Goyang-si, Korea; 2 Department of Molecular Cell Biology, Samsung Biomedical Research Institute, Sungkyunkwan University School of Medicine, Suwon, Korea; 3 Center for Lung Cancer, National Cancer Center, Goyang-si, Korea; 4 Department of Internal Medicine, Samsung Medical Center, Sungkyunkwan University School of Medicine, Seoul, Korea; 5 Department of Pathology, Samsung Medical Center, Sungkyunkwan University School of Medicine, Seoul, Korea; 6 Department of Thoracic and Cardiovascular Surgery, Samsung Medical Center, Sungkyunkwan University School of Medicine, Seoul, Korea; Peking University Health Science Center, China

## Abstract

**Background:**

This study was aimed at investigating the functional significance of heparan sulfate (glucosamine) 3-*O*-sulfotransferase 2 (*HS3ST2*) hypermethylation in non-small cell lung cancer (NSCLC).

**Methodology/ Principal Findings:**

*HS3ST2* hypermethylation was characterized in six lung cancer cell lines, and its clinical significance was analyzed using 298 formalin-fixed paraffin-embedded tissues and 26 fresh-frozen tissues from 324 NSCLC patients. MS-HRM (methylation-specific high-resolution melting) and EpiTYPER^TM^ assays showed substantial hypermethylation of CpG island at the promoter region of *HS3ST2* in six lung cancer cell lines. The silenced gene was demethylated and re-expressed by treatment with 5-aza-2′-deoxycytidine (5-Aza-dC). A promoter assay also showed the core promoter activity of HS3ST2 was regulated by methylation. Exogenous expression of HS3ST2 in lung cancer cells H460 and H23 inhibited cell migration, invasion, cell proliferation and whereas knockdown of HS3ST2 in NHBE cells induced cell migration, invasion, and cell proliferation *in* *vitro*. A negative correlation was observed between mRNA and methylation levels of HS3ST2 in 26 fresh-frozen tumors tissues (ρ = -0.51, *P* = 0.009; Spearman’s rank correlation). *HS3ST2* hypermethylation was found in 95 (32%) of 298 primary NSCLCs. Patients with *HS3ST2* hypermethylation in 193 node-negative stage I-II NSCLCs with a median follow-up period of 5.8 years had poor overall survival (hazard ratio = 2.12, 95% confidence interval = 1.25–3.58, *P* = 0.005) compared to those without *HS3ST2* hypermethylation, after adjusting for age, sex, tumor size, adjuvant therapy, recurrence, and differentiation.

**Conclusions/ Significance:**

The present study suggests that *HS3ST2* hypermethylation may be an independent prognostic indicator for overall survival in node-negative stage I-II NSCLC.

## Introduction

Lung cancer is the leading cause of cancer-related deaths in the world. Despite significant advances in the early detection and treatment in the past two decades, the prognosis remains poor, with the overall 5-year survival rate hovering at less than 15% [1]. The poor prognosis of lung cancer patients results largely from micrometastasis, which makes cure less likely, and partially from the high rate of recurrence after curative resection; patients with stage I lung cancer have a five-year survival of <50% if the cancer has spread to nearby lymph nodes or other areas. More than 80% of recurrences occur within the first two years; recurrence rates after curative surgical resection with appropriate lymph node dissection range from 20% to 50%, depending on the pathologic stage [2]. Thus, the discovery of molecular biomarkers for early detection and identification of patients at high risk of recurrence is clearly important. 

Heparan sulfate (HS) is a linear polysaccharide that is found in all animal tissues, and it occurs as a proteoglycan in which two or three linear heparan sulfate glycosaminoglycan (GAG) chains are covalently attached at specific serine residues on a core protein through a tetrasaccharide linker [3]. The HS chains are assembled on a core protein by enzymes in the Golgi apparatus and are comprised of repeating disaccharide units of alternating glucuronic (GlcA) or iduronic (IdoA) acid and *N*-acetyl glucosamine (GlcNAc). During assembly, they undergo a series of sequential modifications beginning with *N*-deacetylation and *N*-sulfation of the glucosamine residues. Adjacent to this first modification, some GlcA residues are epimerized to iduronic acid by a C5 epimerase and then 2-O-sulfated, with further sulfation at the 6-O and 3-O positions of glucosamine residues to yield mature chains [4,5]. The modifications are important to the fine structure of heparan sulfate proteoglycans (HSPG) and in the specificity of heparan sulfate-protein interaction. *Heparan sulfate* (*glucosamine*) *3-O-*sulfotransferase *2* (*HS3ST2*), a member of the heparan sulfate biosynthetic enzyme family, possesses heparan sulfate glucosaminyl 3-*O*-sulfotransferase activity that modifies GAG chains [6,7]. 

*HS3ST2* hypermethylation has recently been reported in a variety of cancers, such as breast cancer [8,9], colorectal cancer [8,10,11], gastric cancer [12], hematological neoplasm [13], lung cancer [8], pancreatic cancer [8], prostate cancer [14], and cervical cancer [15,16]. *HS3ST2* hypermethylation was found at a high frequency in ductal carcinoma in situ of breast [9] and in cytology specimens of cervical intraepithelial neoplasia 3 (CIN3) [15], suggesting *HS3ST2* hypermethylation probably occurs early during malignant transformation of the breast and cervix. *HS3ST2* also shows high methylation in prostate cancer with recurrence [14]. In addition, the frequency of *HS3ST2* hypermethylation is high in high-grade squamous intraepithelial lesion (HSIL) of the cervix compared to low-grade squamous intraepithelial lesion (LSIL) [16]. However, the clinicopathological significance of *HS3ST2* hypermethylation remains elusive in lung cancer. To gain better insight into the role of *HS3ST2* hypermethylation in non-small cell lung cancer (NSCLC), we characterized *HS3ST2* hypermethylation in six lung cancer cell lines and investigated the effect of hypermethylation of *HS3ST2* on the phenotype and prognosis of lung cancer in paraffin-embedded tissues from 298 primary non-small cell lung cancers (NSCLCs).

## Materials and Methods

### Cell culture and tissue samples

Six human lung cancer cell lines (H23, H226, H460, H520, H1650, and A549) and two human bronchial epithelial cell lines (HBEC and NHBE) were obtained from the American Type Culture Collection (Manassas, VA). The cells were cultured in a designated growth media supplemented with 10% heat-inactivated fetal bovine serum (Hyclone, Logan, UT) and 1% antibiotic-antimycotic (Invitrogen, USA). Twenty-six fresh-frozen tumor and corresponding normal tissues, as well as 298 paraffin-embedded tumor tissues, were obtained from a total of 324 NSCLC patients who underwent curative resection at the Department of Thoracic and Cardiovascular Surgery, Samsung Medical Center, Seoul, Korea between August 1994 and November 2005. This study was approved by the Institutional Review Board at Samsung Medical Center, and written informed consent for the use of tissues was obtained from each patient before surgery. Post-operative follow-up for survival or recurrence was conducted as previously described [2]. Pathological TNM stage was determined according to the guidelines of The American Joint Committee on Cancer [17].

### Genomic DNA extraction and sodium bisulfite modification

H&E (Hematoxylin and Eosin) staining of fresh-frozen and paraffin-embedded tissues was performed; the tumor areas contained at least 75% neoplastic tissue. Genomic DNA from cultured cells and from 26 fresh-frozen tumor and corresponding normal tissues and 298 paraffin-embedded tumor tissues was extracted using the MagAttract DNA Mini Kit (Qiagen, Hilden, Germany) and DNeasy Tissue kit (Qiagen, Valencia, CA), respectively. One microgram of genomic DNA was modified by sodium bisulfite using the EZ DNA Methylation-Gold Kit (ZYMO Research, Irvine, CA). 

### MS-HRM assay, EpiTYPER^TM^ assay, and methylation-specific PCR

**Figure 1:**
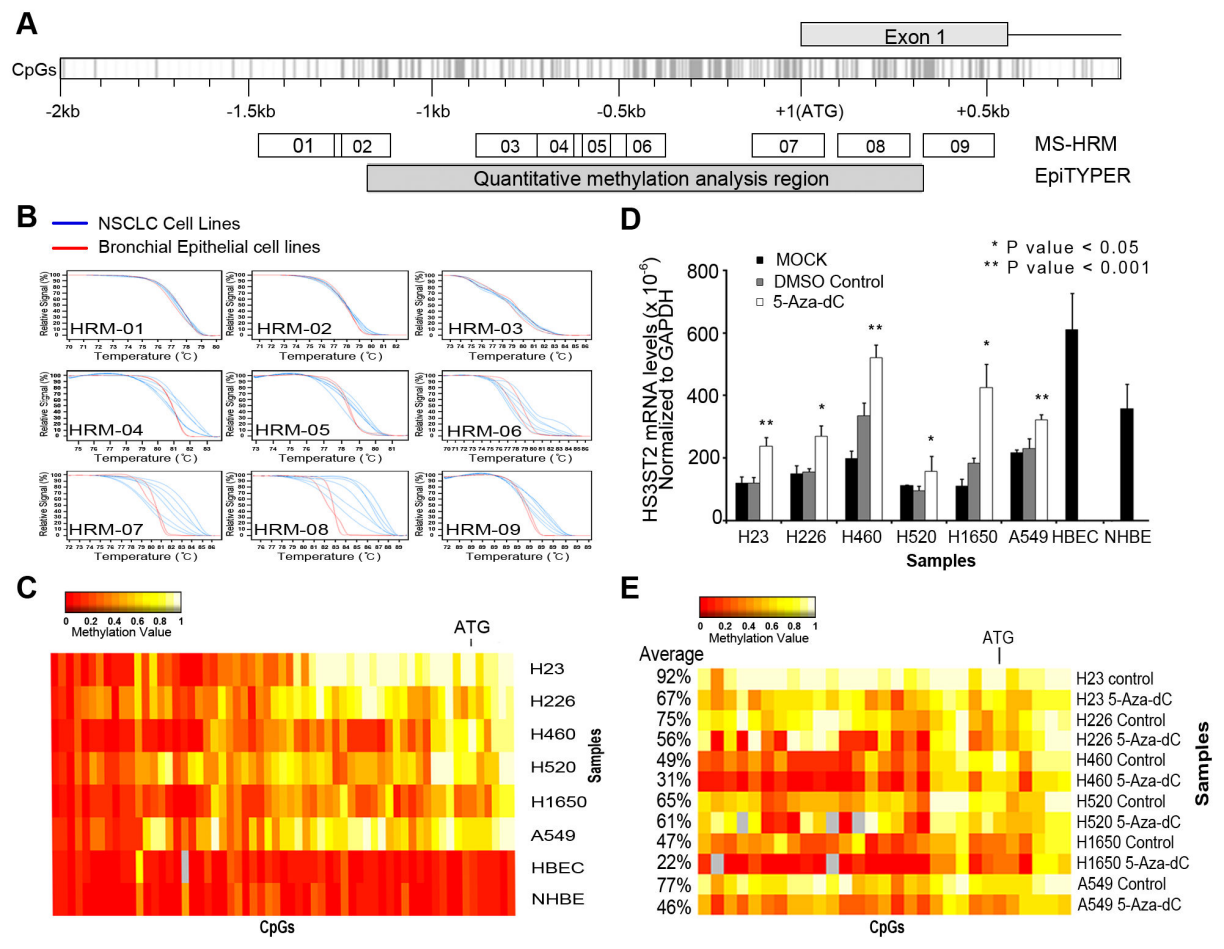
Expression recovery of silenced *HS3ST2* gene by 5-Aza-dC. (A) CpG island map of *HS3ST2* promoter and the regions analyzed using MS-HRM and EpiTYPER assay. (B) Methylation status of *HS3ST2* was first evaluated in six lung cancer cell lines and two bronchial epithelial cell lines using MS-HRM. (C) Methylation status of individual CpGs at a 1.5 kb promoter region (HRM-04, 05, 06, 07, 08, and 09) was quantitatively analyzed in the cell lines using the EpiTYPER^TM^ assay. Methylation levels are depicted as a color change on a continuous scale from red (0% methylated) to white (100% methylated). (D) Transcript levels of *HS3ST2* were compared between highly methylated lung cancer cell lines and bronchial epithelial cell lines by qRT-PCR (black bar). Recovery of *HS3ST2* transcription was assessed after treatment of six lung cancer cell lines with 5-Aza-dC for 72 h (gray bar vs. white bar; *P*-values are based on Wilcoxon signed-rank test). (E) Methylation status of CpGs around the highly methylated ATG area of the *HS3ST2* gene was assessed after treatment with 5-Aza-dC for 72 h. The heatmap shows typical patterns of demethylation in cancer cells in response to 5-Aza-dC, and the percentages indicate average methylation levels.

To find highly methylated regions in the *HS3ST2* promoter, a 2 kb region containing the 1.5 kb *HS3ST2* promoter was analyzed with a methylation-specific high-resolution melt (MS-HRM) assay on a LightCycler®480 real-time PCR instrument (Roche, Switerland). The methylation status of a 1.5 kb region that was found to be highly methylated by MS-HRM assay was further quantitatively analyzed using the EpiTYPER^TM^ assay (Sequenom, San Diego, CA) to analyze methylation statuses of individual CpGs. Analyzed regions are represented in Figure 1A. Primers for each assay were designed using SEQUENOM (San Diego, CA) EpiDesigner software (Table S1 and S2). The methylation status of *HS3ST2* in the 298 formalin-fixed paraffin-embedded tissues was determined using methylation-specific PCR (MSP) with two sets of primers (Table S3) covering the promoter region, as previously described^2^. Reactions were hot started at 94°C before adding 2.5 units of Taq polymerase. Amplification was carried out over 35 cycles (30 s at 94°C, 30 s at the annealing temperature, 30 s at 72°C), followed by 4 minutes at 72°C. The results from the MSP analysis were visually scored by two authors (Y Kim and D-H Kim). Samples with a stronger band intensity than the negative control in the methylation-specific PCR were denoted methylated, and samples with no visible PCR product were regarded as unmethylated. 

### Analysis of HS3ST2 expression

To examine the expression of HS3ST2 at the level of mRNA, cDNA was synthesized from two µg of total RNA using the SuperScript^TM^ III First-Strand Synthesis System (Invitrogen, CA). The mRNA expression of HS3ST2 was analyzed using RT-PCR and quantitative real-time PCR (qRT-PCR). Quantitative real-time PCR was performed on a LightCycler^®^ 480 Real-Time PCR System (Roche Applied Science). GAPDH was used as a reference gene, and the relative expression level was calculated using the ΔC_T_ method (ΔC_T_= C_T_ of GAPDH - C_T_ of HS3ST2). The experiment was performed in triplicate and the qRT-PCR primers used were the same ones as in the RT-PCR. The primers were designed to contain exon-exon junction (Table S3).

### 5-Aza-dC treatment in vitro

Six lung cancer cell lines were incubated with 10 μM 5-aza-2′-deoxycytidine (5-Aza-dC; Sigma Aldrich, St. Louis, MO) for 72 h. Following incubation, mRNA expression and methylation was quantified by quantitative real-time PCR and EpiTYPER^TM^ assay, respectively.

### Reporter assay

Serial deletion constructs of the HS3ST2 promoter were created, and different lengths of promoter regions (-984~+12, -742~+12, -495~+12, -245~+12) were amplified using PCR primers (Table S4) and cloned into the pGL3-basic vector. The promoter sequence was *in vitro* methylated by CpG-specific *Sss*I methylase (New England Biolabs, Ipswich, MA). The promoter activities of methylated and unmethylated constructs were compared by Dual Luciferase assay (Promega, Madison, WI).

### Cell migration, invasion, and proliferation assay

For *in vitro* cell migration, invasion, and proliferation assays, a pAcGFP1-C1-*HS3ST2* cDNA clone was prepared using the primers listed in Table S3, and H460 and H23 cells were transfected with one µg pAcGFP1-C1-*HS3ST2* and pAcGFP1-C1 (empty vector) using Lipofectamine™ 2000 (Invitrogen, USA). Forty-eight hours after transfection, HS3ST2 expression was confirmed by immunoblotting using primary antibodies directed to GFP (sc-9996; Santa Cruz Biotechnology, CA) and α-tubulin (Sigma Aldrich, St. Louis, MO). Cell migration was assayed by allowing cells to migrate downward overnight at 37°C through an 8-m pore filter insert (BD, USA) and then by staining with 1% crystal violet; the dissolved cells were measured at 564 nm in a VERSA_max_ microplate reader (Molecular Devices, USA). The invasion assay was performed by counting Diff-Quick^TM^ (Sysmex, Japan) stained cells in a Matrigel-coated insert following a 48 h incubation. For the proliferation assay, wells were seeded with 1 × 10^3^ cells per well and an MTT assay was performed every 24 h; proliferating cells were measured by absorbance at 570 nm. 

### Knockdown of HS3ST2

HS3ST2-specific small interfering RNA (siRNA target sequence: 5’-CCCAGCTACTTTGTCACTCAA-3’ Cat. No. SI00442015, Qiagen) or negative control siRNA (Cat.No. 10272810, Qiagen) was transfected to NHBE cells. After 72 h of transfection, silencing of HS3ST2 was confirmed by immonublotting using primary antibodies directed to HS3ST2 (Cat. No. PA5-26522, Thermo SCIENTIFIC) and normalized with β-actin. The effect of HS3ST2 knockdown on cell migration, cell invasion and cell proliferation was analyzed as described above.

### Quantitative methylation-specific PCR

Methylation status of CpG island at the promoter region of *HS3ST2* in 26 fresh-frozen tumor tissues was quantitatively analyzed using fluorescence-based quantitative real-time PCR assay, MethyLight, as described by Gonzalo et al [10]. Briefly, the MethyLight reactions were carried out in a final volume of 12.5 µl containing 1.0 µl bisulfite-treated DNA, 900 nM each primer and 250 nM TaqMan probe. Thermocycling reactions were run using a 7300 Real Time PCR System (Applied Biosystems, Foster City, CA), and the *ALU-C4* gene was used as an internal reference to normalize the amount of input DNA [18]. The primers and fluorescence-labeled TaqMan probes used in the present study are presented in Table S5.

### Immunohistochemistry of Ki-67

Immunohistochemical analysis of Ki-67 was performed as described previously [19]. The fraction of Ki-67-positive cells (the Ki-67 labeling index) was defined as the percentage of nuclei stained positively with the antibody.

### Statistical analysis

The Wilcoxon rank sum test (or *t*-test) and Fisher’s exact test (or the Chi-squared test) were used for univariate analysis of the continuous and categorical variables, respectively. HS3ST2 mRNA levels between tumor tissues and matched normal tissues were compared using the Wilcoxon signed-rank test. Spearman’s rank correlation coefficient was calculated for the evaluation of the correlation between expression and methylation of *HS3ST2*. The effect of *HS3ST2* hypermethylation on survival or recurrence was estimated by Kaplan-Meier survival curves, and the significance of differences in prognosis between groups was evaluated by the log-rank test. Cox proportional hazards analysis was performed to estimate the hazard ratios of *HS3ST2* hypermethylation for survival, after controlling for potential confounding factors.

## Results

### Analysis of methylation around the HS3ST2 promoter

The methylation status of *HS3ST2* around the *HS3ST2* promoter was analyzed using MS-HRM (Figure 1B) and EpiTYPER^TM^ assays (Figure 1C) in six lung cancer cell lines and two normal bronchial epithelial cell lines. Methylation status of a 2.0 kb region containing the promoter sequence of *HS3ST2* was first screened by MS-HRM. Notable melting curve dissociation was observed in six fragments (HRM-04, 05, 06, 07, 08, and 09) between lung cancer cell lines and normal bronchial epithelial cell lines (Figure 1B). Because methylated cytosine requires a higher melting temperature than unmethylated cytosine, the melting curves of the methylated fragments shifted to the right in comparison to normal cell lines. To evaluate the methylation statuses of individual CpGs at the 1.5 kb promoter region including the six fragments, we quantitatively analyzed individual CpGs with the EpiTYPER^TM^ assay (Figure 1C). Normal cell lines were hypomethylated in whole 1.5 kb sequences, but lung cancer cell lines were usually hypermethylated around the ATG translation start site. Although some CpGs at the ATG translation start site of *HS3ST2* were partially methylated in H226 and H1650 cells, most CpGs were highly methylated in other lung cancer cell lines. 

### 5-Aza-dC induced demethylation and re-expression of silenced HS3ST2

To investigate if downregulation of *HS3ST2* is dependent on hypermethylation of the gene, we analyzed the re-expression (Figure 1D) and demethylation (Figure 1E) of silenced *HS3ST2* by qRT-PCR and EpiTYPER^TM^ assay after treatment of lung cancer cells with 10 μM 5-Aza-dC for 72 h. HS3ST2 mRNA levels were substantially lower in six lung cancer cell lines than in two bronchial epithelial cell lines (black bar; Figure 1D). Silenced *HS3ST2* was re-expressed in all lung cancer cell lines after 5-Aza-dC treatment (white bar; Figure 1D). The change in the level of methylation in response to 5-Aza-dC was further analyzed at individual CpGs around the highly methylated ATG area of the *HS3ST2* gene (Figure 1E). Although the degree of demethylation differed amongst cell lines, demethylation was found in all cell lines following 5-Aza-dC treatment. Demethylation occurred more substantially in H460 or H1650 cells, which were less densely methylated, than in densely methylated H23 cells. These findings suggest that *HS3ST2* hypermethylation may be associated with transcriptional downregulation of the gene. 

### *HS3ST2* promoter activity decreased with promoter methylation

**Figure 2:**
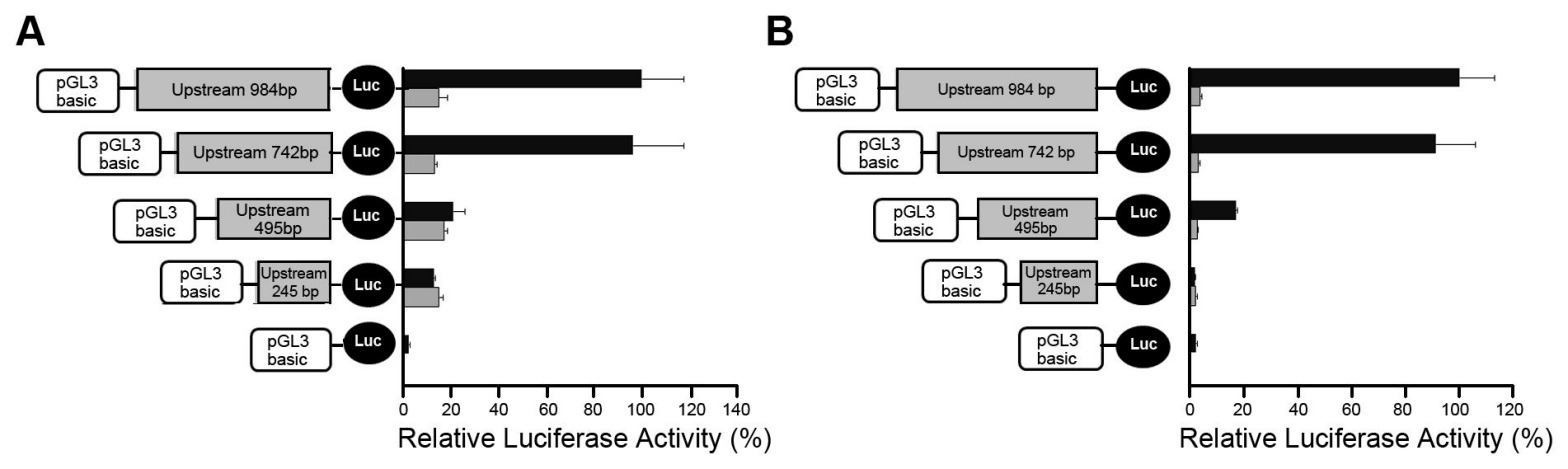
Reporter assay. The dual luciferase activities of four HS3ST2 promoter constructs were measured in H23 (A) and HEK-293T (B) cell lines. One kb promoter construct was serially deleted, and luciferase activity of each construct was compared between constructs treated with (gray bar) and without SssI methylase (black bar).

Promoter activity was measured in H23 cells (Figure 2A) and HEK-293T cells (Figure 2B) by the Dual Luciferase assay to determine if *HS3ST2* methylation affects gene transcription. Four constructs (-984~+12, -742~+12, -495~+12, -245~+12) for each methylated and unmethylated promoter sequences were produced. Methylated constructs of *HS3ST2* promoter were obtained with SssI methylase (New England Biolabs, Inc., Beverly, MA) in the presence of S-adenosylmethionine. Promoter activity among unmethylated constructs of *HS3ST2* promoter was not proportional to the length of each construct in the cells, but the promoter activity was notably decreased when promoter sequences between -742 bp and -495 bp were deleted. For methylated constructs, promoter activity was similar in all constructs and substantially low compared to that of corresponding unmethylated constructs. This observation suggests that the core promoter encompasses a DNA region of -742 bp through -495 bp from the transcription initiation site.

### HS3ST2 inhibited cell migration, invasion, and proliferation

**Figure 3:**
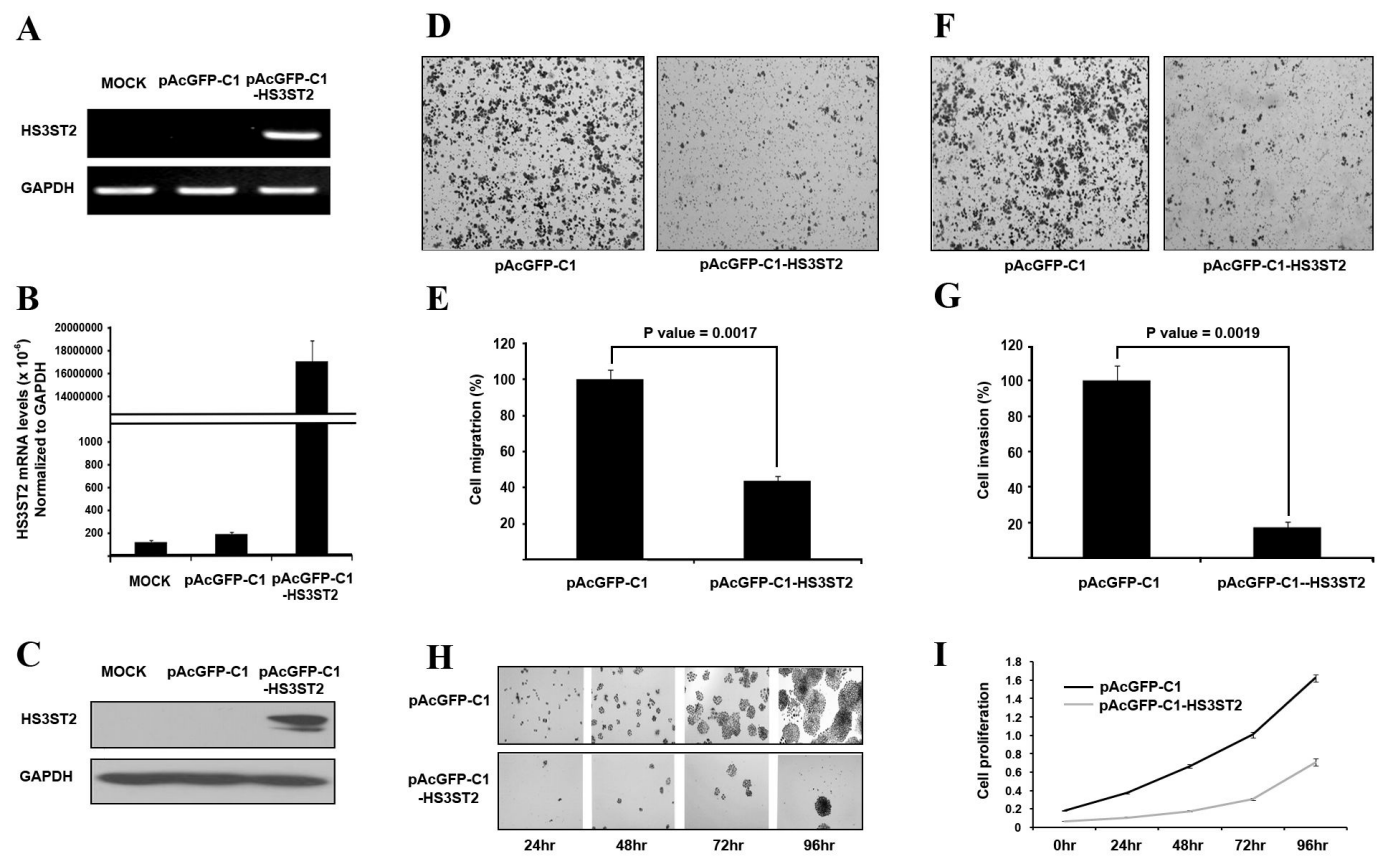
Induced expression of *HS3ST2* inhibited cell migration, invasiveness, and proliferation. (**A**-**C**) pAcGFP-C1-*HS3ST2* was transfected into H460 lung cancer cells. Specific overexpression of *HS3ST2* compared to the empty vector was confirmed by RT-PCR (A), qRT-PCR (B), and western blotting (C). (D-G) H460 cells were seeded in a Boyden chamber with an empty vector or with one μg pAcGFP-C1-*HS3ST2* in transwell migration (D & E) and invasion assays (F & G) as described in the Materials and Methods. Stained migrating cells and invading cells are shown in (D) and (F), respectively; migrating cells were measured by absorbance at 564 nm; invading cells were counted under a microscope. The results are presented relative to the *in* *vitro* migration or invasion of uninduced H460 cells (100%). (H & I) H460 cells transfected with an empty vector or pAcGFP-C1-*HS3ST2* were cultured in a 96-well dish to analyze the effect of *HS3ST2* on cell growth. Cell proliferation was measured by MTT assay and absorbance was measured at 570 nm. Data are presented as the mean ± standard error (SE) of triplicate experiments.

To investigate the function of *HS3ST2* in tumorigenesis of the lung, cell migration, invasion, and proliferation were examined in H460 and H23 lung cancer cells. *HS3ST2* cDNA was cloned into pAcGFP-C1 and transfected into H460 and H23 cells: data of H23 cells are in Figure S1. Overexpression of transfected pAcGFP-C1-*HS3ST2* was monitored by RT-PCR (Figure 3A), qRT-PCR (Figure 3B), and immunoblotting (Figure 3C). Overexpression of *HS3ST2* significantly reduced cell migration capacity by 53% (*P* = 0.0017; Figures 3D and 3E). Cell invasion activity decreased by 83% in H460 cells transfected with pAcGFP-C1-*HS3ST2* (*P* = 0.0019; Figures 3F and 3G); cell proliferation also substantially decreased over time (Figures 3H & 3I). These observations suggest that *HS3ST2* may inhibit lung tumorigenesis by reducing cell migration, invasion, or cell proliferation in lung cancer.

### Knockdown of HS3ST2 increased cell migration, cell invasion and cell proliferation

**Figure 4:**
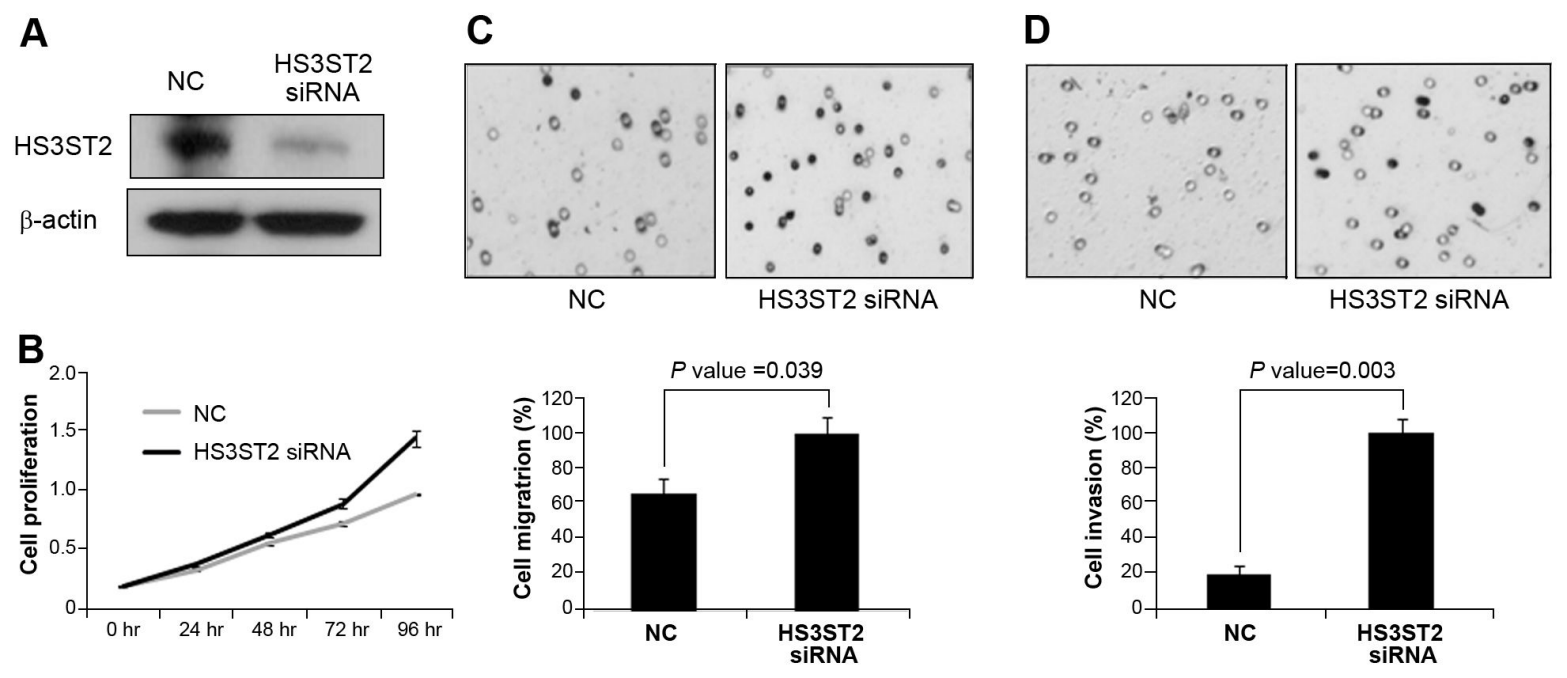
Knockdown of *HS3ST2* expression induced cell migration, cell invasion and cell proliferation. (A) HS3ST2 siRNA was transfected into NHBE cells, and siRNA-mediated knockdown was confirmed by Western blots. (B) Cell viability was measured by MTT assay determined by evaluating the absorbance of the converted dye at a wavelength of 570 nm. (C & D) The effect of HS3ST2 knockdown on cell migration (C) and invasion (D) was also measured in NHBE cells. NC indicates untreated normal control.

To confirm the data of HS3ST2 ectopic expression, the effects of *HS3ST2* knockdown on cell migration, invasion, and cell proliferation were analyzed in NHBE bronchial epithelial cells. After 72h of *HS3ST2* knockdown, silencing was confirmed by immunoblotting (Figure 4A). Knockdown of *HS3ST2* caused an increase in cell proliferation (Figure 4B). Cell migration and invasion also increased by 45% and 79% in silenced NHBE cells, respectively (Figures 4C & 4D). 

### Relationship of clinicopathological characteristics with HS3ST2 hypermethylation

**Figure 5:**
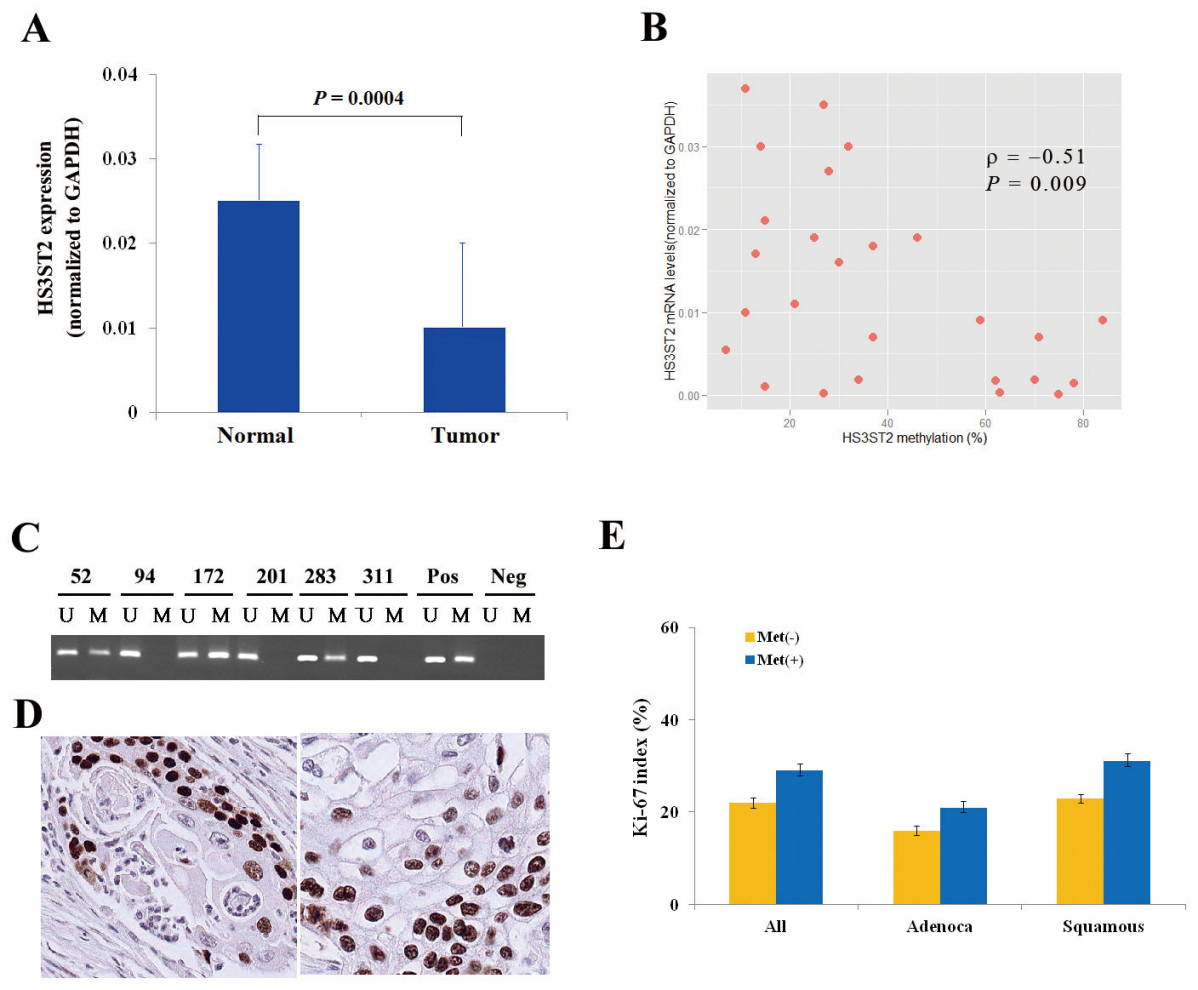
Relationship between *HS3ST2* hypermethylation and clinicopathological variables. (**A**) HS3ST2 mRNA levels were first compared quantitatively between 26 fresh-frozen tumor tissues and corresponding normal tissues (Wilcoxon signed-rank test, *P* = 0.0004). Data are presented as the mean 2^-ΔC^_T_ value of triplicate experiments, and the bars represent standard deviations. (B) Negative correlation between expression and methylation levels of *HS3ST2* was observed using Spearman’s rank correlation analysis in 26 tumor tissues (ρ = -0.51, *P* = 0.009). (C) Methylation of the *HS3ST2* promoter was analyzed in paraffin-embedded tissues from NSCLC patients using methylation-specific PCR (MSP). Patient identification numbers are indicated. “M” and “U” represent amplification of the methylated and unmethylated allele, respectively. “Pos” represents the positive controls for the methylated and unmethylated alleles. Negative control samples without DNA were included for each PCR. (D) Expression of Ki-67 was analyzed by immunohistochemical analysis. Figures show representative examples of positive expression of Ki-67 in adenocarcinoma (left) and squamous cell carcinoma (right) (Magnification: ×200). (E) Association of *HS3ST2* hypermethylation with Ki-67 proliferation index was evaluated in 298 NSCLCs. Error bars represent standard error. “Adenoca” and “Squamous” indicate adenocarcinoma and squamous cell carcinoma, respectively. “Met (+)” and “Met (-)” represent the presence and absence of *HS3ST2* hypermethylation, respectively.

Before analyzing the clinicopathological significance of *HS3ST2* hypermethylation in NSCLC, the relationship between *HS3ST2* hypermethylation and its expression was analyzed in fresh-frozen tissues. Quantitative real-time PCR was performed in fresh-frozen tumor and matched normal tissues from only 26 patients due to the small amount of tissues, and quantitative methylation-specific PCR was conducted in tumor tissues from the patients. HS3ST2 mRNA levels were significantly higher in normal tissues than in tumor tissues (*P* = 0.0004, Wilcoxon signed-rank test; Figure 5A), and negative correlation was observed between expression and methylation of the *HS3ST2* in 26 tumor tissues (ρ = -0.51, *P* = 0.009, Spearnman’s rank correlation; Figure 5B). The methylation status of *HS3ST2* was analyzed in 298 formalin-fixed paraffin-embedded tissues using methylation-specific PCR (MSP) (Figure 5C). The relationships between clinicopathological characteristics and *HS3ST2* hypermethylation are described in Table 1. *HS3ST2* hypermethylation was found in 95 (32%) of 298 patients. *HS3ST2* hypermethylation was not associated with patient age (*P* = 0.12), tumor size (*P* = 0.14), sex (*P* = 0.33), histology (*P* = 0.12), differentiation (*P* = 0.31), pathologic stage (*P* = 0.66), or tumor recurrence (*P* = 0.20). However, *HS3ST2* hypermethylation was significantly associated with pack-years of smoking (*P* = 0.01) and was more prevalent in current-smokers than in never-smokers (*P* = 0.002). 

**Table 1:** Clinicopathological characteristics (N=298).

		*HS3ST2* hypermethylation		
Category	Subcategory	No (N=203)	Yes (N=95)	*P*-value
Age^a^		62 ± 8	60 ± 11	0.12
Pack-years^a^		26 ± 24	32 ± 25	0.01
Size (cm)^a^		3.9 ± 1.9	4.3 ± 2.1	0.14
Sex	Women	45	26	
	Men	158	69	0.33
Smoking status	Never	73	15	
	Former	32	17	
	Current	98	63	0.002
Histology	Adeno^b^	77	48	
	Squamous^b^	107	39	
	Others	19	8	0.12
Differentiation^c^	Well	25	17	
	Moderately	95	37	
	Poorly	29	18	
	Undifferentiated	7	2	0.31
Pathologic stage	I	118	58	
	II	53	19	
	III	30	17	
	IV	2	1	0.66
Recurrence	No	110	59	
	Yes	93	36	0.20

a Mean ± standard deviation

b Adeno, adenocarcinoma; squamous, squamous cell carcinoma

c Differentiation data are missing in 68 cases

To analyze the relationship of *HS3ST2* hypermethylation to cell proliferation, Ki-67 expression was evaluated by immunohistochemical staining (Figure 5D). The Ki-67 proliferation index was found to be significantly different according to the methylation status of *HS3ST2* (*P* = 0.02; Figure 5E): the mean Ki-67 proliferation index was 29% and 22% in tumors with and without *HS3ST2* hypermethylation, respectively. The relationship between the Ki-67 proliferation index and *HS3ST2* hypermethylation was also similar in adenocarcinoma (*P* = 0.04) and squamous cell carcinoma (*P* = 0.009).

### Survival analysis

**Figure 6:**
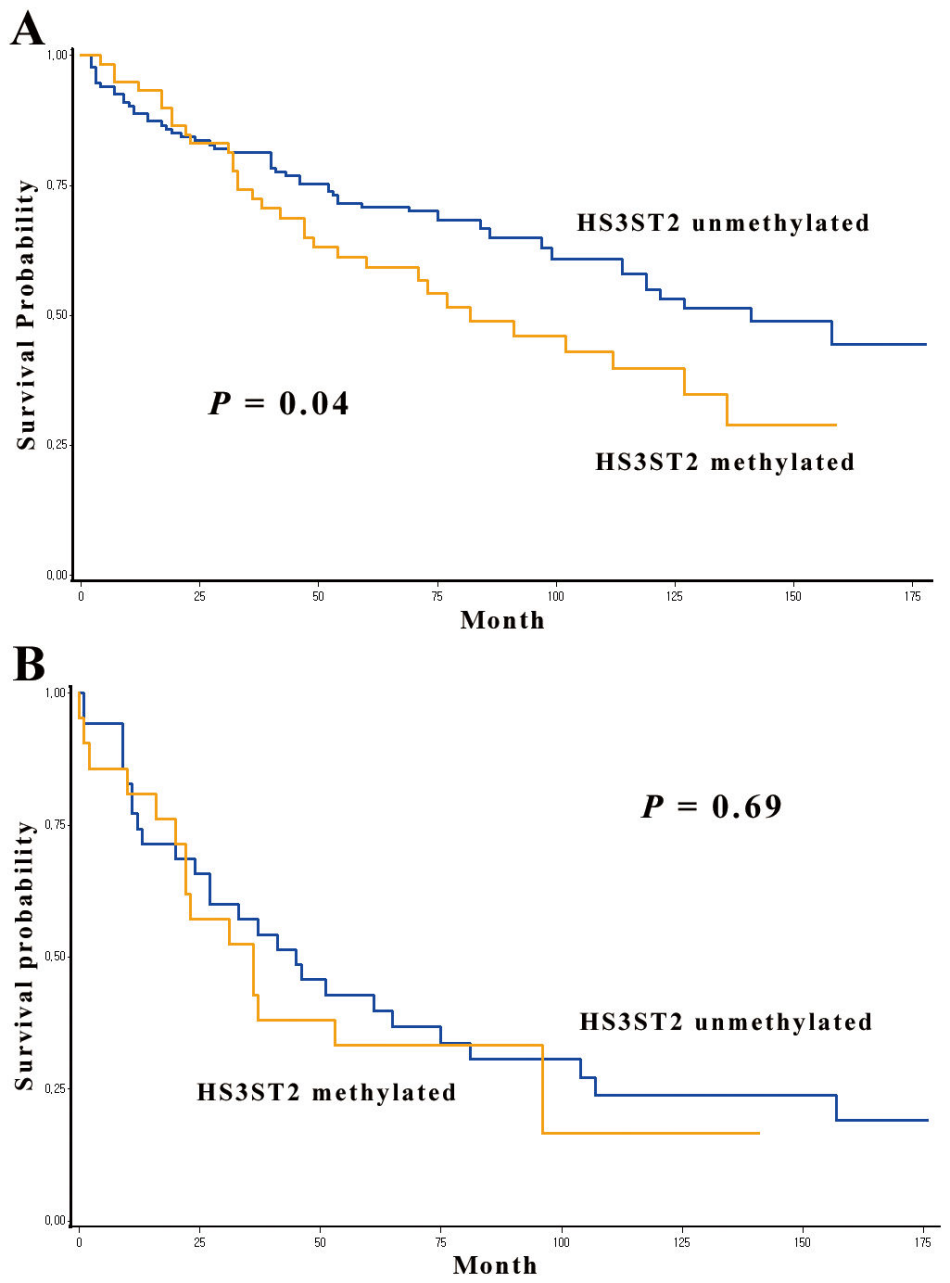
Kaplan-Meier survival curves for *HS3ST2* hypermethylation in stage I-II NSCLCs. Overall survival was compared according to methylation status of *HS3ST2* in 193 node-negative stage I-II NSCLCs (A) and in 55 node-positive stage I-II NSCLCs (B). *HS3ST2* hypermethylation in a group that do not have involvement of lymph node showed a significant adverse effect on overall survival (*P* = 0.04).

Recurrence-free survival (RFS) and overall survival were compared according to methylation status of *HS3ST2* across tumor stage. To analyze the effect of *HS3ST2* hypermethylation on patient prognosis, we stratified data by tumor stage because tumor stage is significantly associated with patient survival in NSCLC. *HS3ST2* hypermethylation was not associated with RFS (data not shown). However, the effect of *HS3ST2* hypermethylation on overall survival showed a different pattern according to involvement of lymph node in 248 stage I-II NSCLCs. For 193 node-negative stage I-II NSCLCs, the 5-year survival rate was 59% and 71% in patients with and without *HS3ST2* hypermethylation, respectively (Figure 6A). And, this difference was significantly different (*P* = 0.04). However, overall survival was not significantly different between patients with and without hypermethylation of *HS3ST2* in 55 node-positive cases (*P* = 0.69; Figure 6B).

### Cox-proportional hazards analysis

Stratified Cox proportional hazards analysis (Table 2) for 248 stage I-II NSCLCs were performed to control for potential confounding effects of variables, such as age, sex, tumor size, adjuvant therapy, recurrence, and differentiation, and to calculate the hazard ratio (HR). Patients with *HS3ST2* hypermethylation in 193 node-negative NSCLCs had a 2.12 times (95% confidence interval [CI] = 1.25 - 3.58; *P* = 0.005) greater risk of failure compared to those without *HS3ST2* hypermethylation. However, overall survival in patients with the involvement of lymph node was not significantly different according to the methylation status of *HS3ST2* (HR = 0.84, 95% CI = 0.34 - 3.82; *P* = 0.69).

**Table 2:** Cox proportional hazards analysis of overall survival in stage I-II.

	*HS3ST2* hypermethylation	HR^a^	95% CI^a^	*P*-value
(A) Node-positive cases	No	1.00		
(N=55)	Yes	0.84	0.34–3.82	0.69
(B) Node-negative cases	No	1.00		
(N=193)	Yes	2.12	1.25–3.58	0.005

Adjusted for age, sex, tumor size, adjuvant therapy, recurrence, and differentiation

a HR, hazard ratio; CI, confidence interval

NSCLCs (N = 248).

## Discussion

The *HS3ST2* gene encodes heparin sulfate glucosamine 3-*O*-sulfotransferase 2, which is known to be involved in the final modification step (3-*O*-sulfation of glucosamine residues of GAG chains), during biosynthesis of heparan sulfate. This modification is important in determining the specificity of the heparan sulfate proteoglycan (HSPG) binding with various proteins and for the regulatory properties of HSPGs. In this study, *HS3ST2* was hypermethylated in 95 (32%) of 298 tumor tissues and associated with poor overall survival in node-negative stage I-II NSCLC. However, it is not clear how *HS3ST2* hypermethylation affects lung tumorigenesis and patient prognosis.

HSPGs are known to be involved in cell growth, adhesion, migration, tumor invasion, and metastasis. The ligands, such as the acidic and basic fibroblast growth factor [20], vascular endothelial growth factor [21], and hepatocyte growth factor [22], use HSPGs as co-receptors to mediate binding to specific tyrosine kinase receptors and promote signal transduction [23]. HSPGs also participate in cell-cell adhesion by binding to structural proteins in the extracellular matrix, including collagen, laminin, and fibronectin. In this study, transfection of pAcGFP-C1-*HS3ST2* fusion protein inhibited cell proliferation, cell migration, and invasion *in vitro* (Figure 4). In addition, tumors with *HS3ST2* hypermethylation showed a significantly higher Ki-67 level than those without (29% vs. 22%; *P* = 0.02), thereby supporting the anti-proliferative effect of *HS3ST2 in vitro*. Based on these observations, it is likely that *HS3ST2* hypermethylation contributes to lung tumorigenesis by altering the interaction of HSPGs with a variety of proteins participating in cell growth, cell adhesion, or other tumorigenic process.

In addition to tumorigenesis of the lung, *HS3ST2* hypermethylation may be involved in patient prognosis. Over 90% of cancer-associated patient mortality is attributable to tumor metastasis because of its systemic nature and the resistance of disseminated cancer cells to therapeutic agents. Although *HS3ST2* hypermethylation in this study was not associated with the M stage (*P* = 0.89) and the prevalence of *HS3ST2* hypermethylation was not significantly different between 193 node-negative cases and 55 node-positive cases (31% vs. 33%, respectively; *P* = 0.82), *HS3ST2* hypermethylation was found to be significantly associated with poor overall survival in patients with node-negative stage I-II NSCLC, but not with node-positive cancer. These observations suggest that *HS3ST2* hypermethylation may contribute to the poor overall survival by another mechanism rather than by lymphatic or systemic spread of cancer cells. Further study is required to understand the molecular mechanisms involved in this process. 

It is not clear what is responsible for *HS3ST2* hypermethylation in NSCLC. Increased activity of DNA methyltransferase is one factor that may be responsible for increased susceptibility to aberrant methylation of CpG islands in the promoter region of tumor suppressor genes [24,25]. Smoking increases the activity of DNA methyltransferase, and thereby induces *de novo* methylation. Increased levels of DNMT1 were observed in the lungs of A/J mice exposed to the tobacco-specific carcinogen 4-methylnitrosamino-1-(3-pyridyl)-1-butanone (NNK) [26]. In addition, several groups have reported a positive relationship between exposure to tobacco smoke and CpG island hypermethylation in NSCLCs, suggesting that hypermethylation of CpG islands may be regulated by smoking [27–29]. Therefore, we analyzed the association between exposure to tobacco smoke and *HS3ST2* hypermethylation in 298 NSCLC patients. In this study, *HS3ST2* did not show age-related hypermethylation, but the risk of *HS3ST2* hypermethylation significantly increased with exposure to tobacco smoke. 

This study was limited by several factors. First, the effect of *HS3ST2* hypermethylation on overall survival needs to be validated by a prospective study to understand its utility as a true prognostic and predictive biomarker that may be reliably applied in a clinical setting. Second, the number of patients with node-positive stage I-II NSCLC who participated in this study was very small. Third, studies in premalignant tissues will clarify the role of *HS3ST2* hypermethylation in lung tumorigenesis. Fourth, in spite of the anti-proliferative effect of HS3ST2 *in vitro*, the tumor size was not significantly different between patients with *HS3ST2* hypermethylation and those without (4.3 cm vs. 3.9 cm, respectively; *P* = 0.14). Finally, considering the high prevalence of *HS3ST2* hypermethylation in stage I cases and the lack of association between *HS3ST2* hypermethylation and pathologic stage, *HS3ST2* hypermethylation may occur at an early stage of the carcinogenic process. Accordingly, further study with a larger sample and in premalignant tissues is needed to solve these limitations. In conclusion, the present study suggests that *HS3ST2* hypermethylation may be associated with poor overall survival in patients with node-negative stage I-II NSCLC. Thus, node-negative stage I-II NSCLC with *HS3ST2* hypermethylation may warrant aggressive treatment after surgery.

## Supporting Information

Figure S1 **The effect of HS3ST2 ectopic expression on cell migration, invasion, and proliferation.** H23 lung cancer cells were transfected by pAcGFP-C1-*HS3ST2*, and ectopic expression of HS3ST2 was confirmed by RT-PCR (A), qRT-PCR (B), and western blotting (C). (D-H)The effect of HS3ST2 on cell proliferation, migration, and invasion was analyzed as described in the Materials and Methods. Ectopic expression of HS3ST2 in H23 cells significantly inhibited cell migration (D & E; *P* = 0.028) and invasion (F & G; *P* = 0.013). In addition, cell proliferation also decreased substantially by HS3ST2.
(TIF)

Table S1 (DOCX)

Table S2 (DOCX)

Table S3 (DOCX)

Table S4 (DOCX)
