# Correction: Epigenetic Inactivation of Heparan Sulfate (Glucosamine) 3-*O*-Sulfotransferase 2 in Lung Cancer and Its Role in Tumorigenesis

**DOI:** 10.1371/journal.pone.0092350

**Published:** 2014-03-24

**Authors:** The PLOS ONE Staff

There are multiple errors in this article.

In the last sentence of the second paragraph of the Introduction section, “sulfotransferase 2” should be italicized.

There are two errors in the "MS-HRM assay, EpiTYPER assay, and methylation-specific PCR" section of the Materials and Methods.

•In the first sentence, the 480 real-time PCR instrument should be listed as the 480II real-time PCR instrument.

•The fifth sentence should refer to reference 2. The correct sentence reads: The methylation status of HS3ST2 in the 298 formalin-fixed paraffin-embedded tissues was determined using methylationspecific PCR (MSP) with two sets of primers (Table S3) covering the promoter region, as previously described [2].

There are two errors in the "Cell migration, invasion, and proliferation assay" section of the Materials and Methods.

•In the first sentence, pAcGFP1 is listed incorrectly three times. All instances should read pAcGFP. The correct sentence is: For in vitro cell migration, invasion, and proliferation assays, a pAcGFP-C1-HS3ST2 cDNA clone was prepared using the primers listed in Table S3, and H460 and H23 cells were transfected with one µg pAcGFP-C1-HS3ST2 and pAcGFP-C1 (empty vector) using Lipofectamine 2000 (Invitrogen, USA).

•In the third sentence, the pore filter insert is incorrectly listed as 8-m instead of 8µm.

In the Figure 1A legend, the EpiTYPER assay should be listed as the EpiTYPER assay.

The first sentence of the "Analysis of methylation around the HS3ST2 promoter" portion of the Results is incorrect. The correct sentence reads: The methylation status around the *HS3ST2* promoter was analyzed using MS-HRM (Figure 1B) and EpiTYPER assays (Figure 1C) in six lung cancer cell lines and two normal bronchial epithelial cell lines.

The Figure 2 legend incorrectly refers to the HEK-293T cell line. The cell line is HEK-293. The correct legend reads: The dual luciferase activities of four HS3ST2 promoter constructs were measured in H23 (A) and HEK-293 (B) cell lines. One kb promoter construct was serially deleted, and luciferase activity of each construct was compared between constructs treated with (gray bar) and without SssI methylase (black bar).

The first sentence of the "*HS3ST2* promoter activity decreased with promoter methylation" section of the Results incorrectly refers to the HEK-293T cell line. The cell line is HEK-293. The correct sentence reads: Promoter activity was measured in H23 cells (Figure 2A) and HEK-293 cells (Figure 2B) by the Dual Luciferase assay to determine if *HS3ST2* methylation affects gene transcription.

In the Author Contributions section, Yong Gu Cho (YGC) should be listed as one of the persons who performed the experiments.
