# Supplementary figures and images for: Epigenetic Inactivation of Heparan Sulfate (Glucosamine) 3-O-Sulfotransferase 2 in Lung Cancer and Its Role in Tumorigenesis
Source: PLoS One. 2013 Nov 12;8(11):e79634. doi: 10.1371/journal.pone.0079634 (PMC3827134; doi:10.1371/journal.pone.0079634)

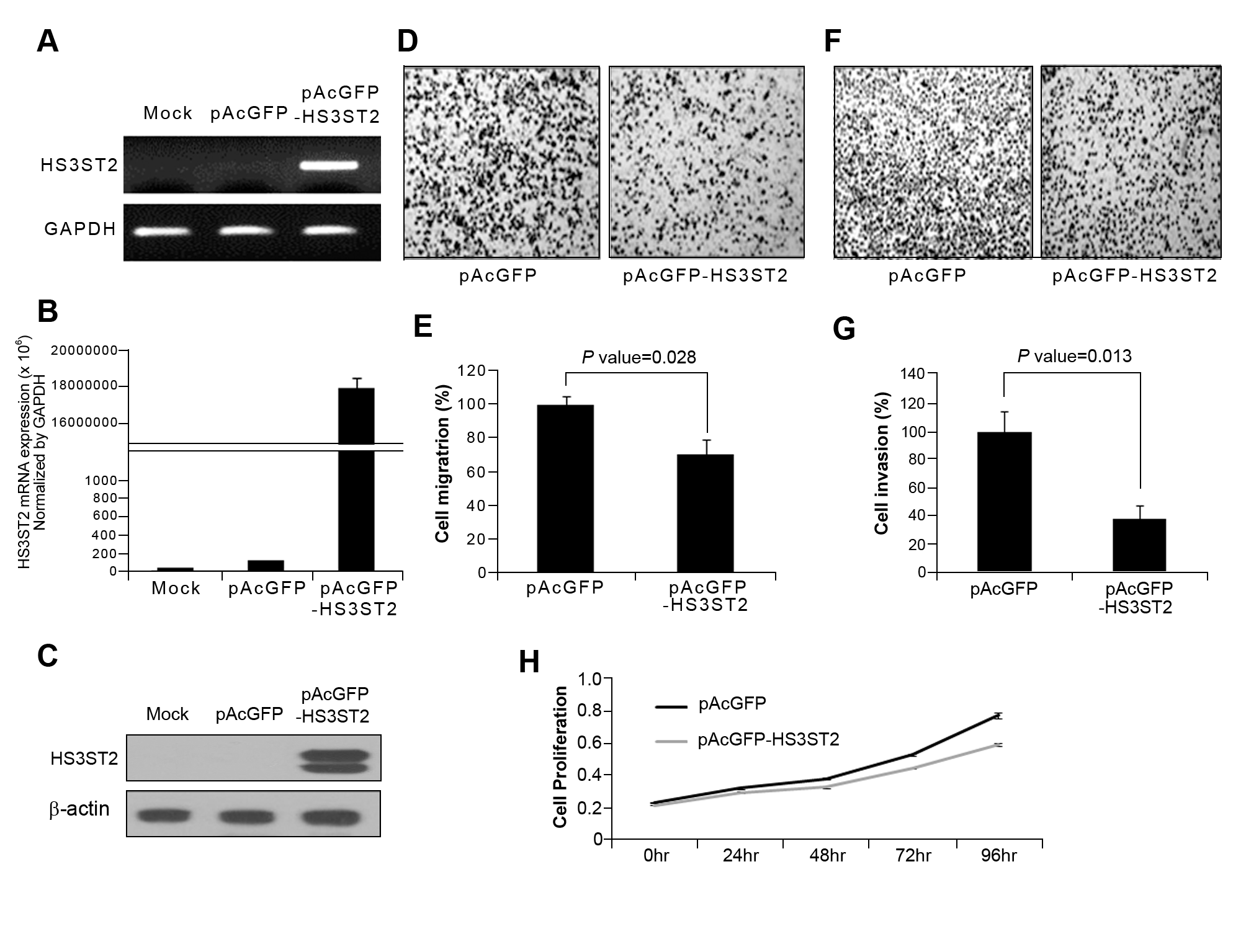

Supplement: Figure S1 — The effect of HS3ST2 ectopic expression on cell migration, invasion, and proliferation. H23 lung cancer cells were transfected by pAcGFP-C1-HS3ST2, and ectopic expression of HS3ST2 was confirmed by RT-PCR (A), qRT-PCR (B), and western blotting (C). (D-H)The effect of HS3ST2 on cell proliferation, migration, and invasion was analyzed as described in the Materials and Methods. Ectopic expression of HS3ST2 in H23 cells significantly inhibited cell migration (D & E; P = 0.028) and invasion (F & G; P = 0.013). In addition, cell proliferation also decreased substantially by HS3ST2. (TIF) [file pone.0079634.s001.tif]
