# Supplementary material for: Epigenetic Inactivation of Heparan Sulfate (Glucosamine) 3-O-Sulfotransferase 2 in Lung Cancer and Its Role in Tumorigenesis
Source: PLoS One. 2013 Nov 12;8(11):e79634. doi: 10.1371/journal.pone.0079634 (PMC3827134; doi:10.1371/journal.pone.0079634)
Supplement: Table S1 — (DOCX) [file pone.0079634.s002.docx]

**Supplementary Table S1. Primer sequences used for MS-HRM**

| Primer ID | Sequence (5'→3') |
| --- | --- |
| HRM-01-F^1^ | TTGATGTAGGGTTTTGATTTAAGGT |
| HRM-01-R^1^ | CTTCTTCACACAAAAACACTAAAAA |
| HRM-02-F | TTTTTTTTAGTGTTTTTGTGTGAAG |
| HRM-02-R | CAAAAAAATTCCTCTCTTAACAACC |
| HRM-03-F | GTATTTGGGTTTAGGTTAGGGGAT |
| HRM-03-R | ACATACAAACCAAACAAACCC |
| HRM-04-F | TTGGGTTTGTTTGGTTTGTATG |
| HRM-04-R | AAAATATCAAAACATAATACTCCTCC |
| HRM-05-F | GGGTTTTTTTTATGTAGGTTATT |
| HRM-05-R | TAATAAAAATATCCAACCTCCC |
| HRM-06-F | GGTTTTATTTAAGGAGTGGTTTGA |
| HRM-06-R | CCCCAAAACTAACCTAAAACTCC |
| HRM-07-F | GGGATTTTTTGGTATTGTG |
| HRM-07-R | CAAAACCCTATAAACCATAACTCCAT |
| HRM-08-F | GGATTTTTGGAGAAGTTTTTGGT |
| HRM-08-R | AAAACCATAACCTATAAAATCCTAAACC |
| HRM-09-F | GGAGTTTTTGGTTTTTATTTGTA |
| HRM-09-R | TTACCCCAAACCCTCATTATAAAC |

^1^F and R indicate forward and reverse, respectively.
