# Supplementary material for: Epigenetic Inactivation of Heparan Sulfate (Glucosamine) 3-O-Sulfotransferase 2 in Lung Cancer and Its Role in Tumorigenesis
Source: PLoS One. 2013 Nov 12;8(11):e79634. doi: 10.1371/journal.pone.0079634 (PMC3827134; doi:10.1371/journal.pone.0079634)
Supplement: Table S2 — (DOCX) [file pone.0079634.s003.docx]

**Supplementary Table S2. Primer sequences used for EpiTyper**

| Primer ID | Sequence (5'→3') |
| --- | --- |
| EpiTYPER-01-10F^1^ | aggaagagagTGTTTTTGTGTGAAGAAGTTTTTTTT |
| EpiTYPER-01-T7R^1^ | cagtaatacgactcactatagggagaaggctAATCCCCTAACCTAAACCCAAAT |
| EpiTYPER-02-10F | aggaagagagGGATTTTTGGAGAAGTTTTTGGT |
| EpiTYPER-02-T7R | cagtaatacgactcactatagggagaaggctCACCCTAATCAACAACCCCC |
| EpiTYPER-03-10F | aggaagagagGGAAGTTGTAATATAGGTAAGTGTAGGAGA |
| EpiTYPER-03-T7R | cagtaatacgactcactatagggagaaggctAAAAAATAACCTACATAAAAAAAACCC |
| EpiTYPER-04-10F | aggaagagagGGAAGTTGTAATATAGGTAAGTGTAGGAGA |
| EpiTYPER-04-T7R | cagtaatacgactcactatagggagaaggctTCAAACCACTCCTTAAATAAAACC |
| EpiTYPER-05-10F | aggaagagagGGGTTTAGGTTAGGGGATTTTTT |
| EpiTYPER-05-T7R | cagtaatacgactcactatagggagaaggctACCCTCCCCATACAAACCAC |

^1^F and R indicate forward and reverse, respectively.
