# Supplementary material for: Epigenetic Inactivation of Heparan Sulfate (Glucosamine) 3-O-Sulfotransferase 2 in Lung Cancer and Its Role in Tumorigenesis
Source: PLoS One. 2013 Nov 12;8(11):e79634. doi: 10.1371/journal.pone.0079634 (PMC3827134; doi:10.1371/journal.pone.0079634)
Supplement: Table S3 — (DOCX) [file pone.0079634.s004.docx]

**Supplementary Table S3. Primer sequences used for MSP, RT-PCR, and cDNA clone**

|  | Forward | Reverse |
| --- | --- | --- |
| **MSP** |  |  |
| Methylated | 5’-TGTTTTTTCGGAAATTATGATTTTC -3’ | 5’- GTAAAAACGAAAAACAACCTACG -3’ |
| Unmethylated | 5’- TTTTTTTGGAAATTATGATTTTTGG -3’ | 5’-AACATAAAAACAAAAAACAACCTACAC-3’ |
| **RT-PCR** |  |  |
| *HS3ST2* | 5’-TGTGGGCGTGAAGAAGGGGG-3’ | 5’-GCTCTCGAGGGTCCTGGGCA-3’ |
| GAPDH | 5-‘TGCACCACCAACTGCTTA-3’ | 5’-GGATGCAGGGATGATGTTC-3’ |
| **cDNA clone** |  |  |
| EcoRI-F^a^ | 5’-CCCGAATTCTGGAGCCATGGCCTATAGGGT-3’ | |
| BamHI-R^a^ | 5’- CCCGGATCCTGGGCTTATTCCCACCTGAAG-3’ | |

^a^F and R indicate forward and reverse primers, respectively.
