# Supplementary material for: Epigenetic Inactivation of Heparan Sulfate (Glucosamine) 3-O-Sulfotransferase 2 in Lung Cancer and Its Role in Tumorigenesis
Source: PLoS One. 2013 Nov 12;8(11):e79634. doi: 10.1371/journal.pone.0079634 (PMC3827134; doi:10.1371/journal.pone.0079634)
Supplement: Table S4 — (DOCX) [file pone.0079634.s005.docx]

**Supplementary Table S4. Quantitative methylation-specific PCR primer and TaqMan probe sequences**

| **Genes** | Forward primer sequence (5'→3') | Probe sequence (5'→3') | Reverse primer sequence (5'→3') |
| --- | --- | --- | --- |
| **HS3ST2** | GTAGTTTTCGGAGAAGACGG | 6FAM- ATTCGCGTGGTCGTG-TAMRA | AACCCTACGATCGCCTAA |
| **ALUC4** | **GGTTAGGTATAGTGGTTTATATTTGTAATTTTAGTA** | 6FAM-CCTACCTTAACCTCCC -TAMRA | **ATTAACTAAACTAATCTTAAACTCCTAACCTCA** |
